# Cytonuclear Genetic Incompatibilities in Plant Speciation

**DOI:** 10.3390/plants9040487

**Published:** 2020-04-10

**Authors:** Zoé Postel, Pascal Touzet

**Affiliations:** University of Lille, CNRS, UMR 8198-Evo-Eco-Paleo, F-59000 Lille, France; zoe.postel@univ-lille.fr

**Keywords:** cytonuclear interactions, intergenomic conflict, reproductive isolation, coevolution

## Abstract

Due to the endosymbiotic origin of organelles, a pattern of coevolution and coadaptation between organellar and nuclear genomes is required for proper cell function. In this review, we focus on the impact of cytonuclear interaction on the reproductive isolation of plant species. We give examples of cases where species exhibit barriers to reproduction which involve plastid-nuclear or mito-nuclear genetic incompatibilities, and describe the evolutionary processes at play. We also discuss potential mechanisms of hybrid fitness recovery such as paternal leakage. Finally, we point out the possible interplay between plant mating systems and cytonuclear coevolution, and its consequence on plant speciation.

## 1. Introduction

Speciation is the evolutionary process that leads to the differentiation of distinct species from an ancestral population. The delimitation of a species can be assessed by the level of barriers to reproduction to another species, i.e., the possibility to mate and produce viable and fertile hybrids. In plants, pre-zygotic barriers can involve differences in phenology, pollinator guild or habitat and pollen-stigma compatibility. Post-zygotic barriers expressed in hybrids can affect germination rate, survival or reproductive traits such as pollen quantity or quality and seed production [1,2,3]. The longer the species have diverged, the more they are expected to be reproductively isolated due to genetic differences, i.e., different fixed substitutions [4]. These mutations might have been directly selected by natural selection, for example if they confer a better adaptation to a given habitat, rendering the hybrid maladapted to both parental habitats. However, more generally, the reduced fitness of hybrids is believed to be due to genetic incompatibilities, i.e., mutations fixed independently by the species, that will interact negatively in the hybrid (Bateson–Dobzhansky–Muller incompatibilities—BDMIs) [5,6]. BDMIs can arise via different evolutionary processes. A given mutation might be neutral or nearly neutral in a given species, fixed by chance (genetic drift), but acting negatively on genes from the other species in the hybrid. If, on the contrary, this first mutation is not neutral and impacts, for instance, possible interactions with other genes, these interactions might be re-established through positive selection. A third possibility is that BDMIs result from a genetic conflict between a selfish element that distorts segregation to increase its own transmission, inducing an arms-race to counteract its effect [5,7,8].

In this review, we will assess how organellar genomes and their interaction with the nucleus can be involved in the process of speciation and reproductive isolation. Interestingly, hybrids from reciprocal crosses often reveal an asymmetry in the level of reproductive isolation. This phenomenon called Darwin’s corollary, points to the possible cytonuclear origin of genetic incompatibilities (cytonuclear incompatibilities, CNIs) [9,10]. In addition, recent studies provide accumulative evidence of the frequent role of cytoplasmic genomes in adaptation and speciation in plants (reviewed in [9,11,12]).

## 2. How Can Cytonuclear Incompatibilities Be Involved in Speciation?

### 2.1. Coevolution between Nuclear and Organellar Genomes

Plastids and mitochondria are essential components of plant cells: mitochondria are responsible for cellular respiration and plastids have an essential role in photosynthesis and seed storage lipid synthesis [12]. The organellar protein complexes are encoded by nuclear and organellar genes, leading to genetic interactions between the nucleus and the organelles [12,13,14,15]. Such a pattern of cytonuclear (CN) interactions is the result of a long-term evolutionary history of organellar endosymbiotic origins [16]. Indeed, both organellar genomes originate from free-living bacteria, integrated into the host cell as endosymbiont, leading to the current eukaryotic cell as we know it [13,16,17]. These endosymbiotic events were followed by a massive functional gene transfer from the endosymbiont genome (i.e., plastidic and mitochondrial) to the host cell (i.e., the nucleus) and a subsequent gene loss of the redundant organellar function, leading to a reduction in size of organellar genomes [16,17]. Thus, organellar genomes do not encode the vast majority of the proteins they need for proper function (e.g., more than 90% of the plastid proteins are encoded by the nucleus [13]). Still, organellar genomes have conserved some key genes from their initial set of genes, encoding subunits (SUs) of enzyme complexes of essential eukaryotic pathways, such as respiration and photosynthesis [16,17,18]. However, for the proper cell performance, these organellar complexes also need gene products from nuclear genes, often derived from former organellar genes transferred to the nucleus [19]. Coordination between organellar and nuclear genomes is thus essential for eukaryotic cells [20] and represents a case of coevolution where organellar and nuclear genomes impose selection pressure on one another for the proper function of plant cells [12,17].

Differences exist between organellar and nuclear genomes. For example, due to the uniparental mode of inheritance resulting in a lack of recombination between parental genomes, organellar genomes have a reduced effective population size (Ne) which reduces the efficacy of natural selection and increases the impact of genetic drift [17]. As an outcome, organellar genomes should be more prone than the nuclear genome to evolve under Muller’s ratchet, i.e., the irreversible accumulation of deleterious mutations due to the lack of recombination [12]. Another contrasting characteristic is the mutation rate, which is much lower in the organellar genomes compared to the nuclear one, at least in land plants [21]. Given the specific features of organellar genomes and the accumulation of deleterious mutations, it is expected that compensatory mutations on the nucleus may be positively selected to maintain coadaptation between interacting genomes and cellular performance [9,12,16,17]. However, the evolutionary dynamics of cytoplasmic genomes and the subsequent cytonuclear co-evolution might be more complex than expected. Analyses of mitochondrial and nuclear genomes in *Drosophila melanogaster* and *Homo sapiens* [22], mammal mitochondrial and proteobacteria genomes [23], suggest that despite their low Ne, mitochondrial genomes exhibit similar efficacy of purifying selection as nuclear ones. This could be explained by a stronger constraint on organellar complexes, due to the high number of protein-protein interactions and/or the additional layer of selection acting on mitochondrial genomes at the individual level, through the reduction of the number of mitochondrial genomes that occurs in the transmission from mother to offspring. A recent theoretical study shows that the way cytoplasmic genomes are transmitted not only slows down the accumulation of deleterious mutations, but also favors the fixation of beneficial ones [24].

#### Detection of Coevolution

Coevolution between two genes can be detected by looking at the encoded polypeptides. When mutual selective pressure exists between these genes, changes of amino acid in one polypeptide will lead to corresponding changes in the other one, resulting in correlated evolutionary rates [25]. Coevolution between genomes can be studied using different methods, based on phylogenies combined with the analysis of the rate of non-synonymous (NS) substitutions (reviewed in [26]). Under the hypothesis of coevolution, NS substitutions of interacting proteins should occur concurrently or sequentially in the phylogeny [15,25].

The generally low rate of evolution of the plastid genome makes it difficult to select a proper set of genes or taxa to study CN coevolution [25,27]. Nevertheless, in some independent lineages, accelerated plastid genome evolution has been detected, leading to faster CN coevolution [16,20,28,29,30]. For example, Geraniaceae exhibit accelerated rates of sequence evolution in plastid genes encoding SUs of the RNA polymerase complex [25]. Evolutionary rates of nuclear genes encoding SUs of this complex were thus estimated [25]. A correlation of the rates of non-synonymous substitutions $\left(d_{N}\right)$ was found between two nuclear genes and all the four plastid genes of this complex [25]. Conversely, no correlation was identified for the rate of synonymous substitution $\left(d_{S}\right)$, suggesting that this correlation is not due to the background mutation rate and very likely reflects coevolution signatures. Sloan et al. (2014) observed fast evolution of the nuclear genes encoding SUs of the organellar ribosomes in *Arabidopsis*, despite a low mutation rate of the organelle genomes [18]. This pattern was even more evident for *Silene* species with an accelerated rate of organellar genome evolution [18]. Interestingly, in these species, nuclear-encoded cytosolic ribosomal proteins, as well as control nuclear genes not involved in organellar function, did not show any sign of accelerated evolution. This suggests that the accelerated rate of organellar genome evolution and the subsequent compensatory mutation in the nuclear compartment resulted in accelerated rates of sequence evolution of the nuclear genes involved in organellar protein complexes [18].

Evidence of coevolution can also be assessed by searching for signatures of positive selection on one of the interactors, i.e., the genes that are involved in the interaction. In response to mutations in the sequence of one interactor (generally, the organellar one), compensatory mutations will arise in the other (generally the nuclear one) and will be positively selected, as it allows the maintenance of the coadaptation and proper cell function. Thus, detecting positive selection in the sequence of nuclear genes encoding SUs of organellar protein complexes can be a sign of coevolution. The detection of positive selection is possible by analyzing the ratio $d_{N}/d_{S}$, which is used as a measure of selection (Box 1). The study of coevolution between plastid and nuclear genes was conducted on two plastid complexes (not involved in the photosynthetic process): Clp and ACCase. Clp complex is required for proper plastid function as it stabilizes the plastome (i.e., plastid genome), but its precise function remains unclear. ACCase is involved in fatty acid biosynthesis. Both complexes are composed of one plastid-encoded SU and several nuclear-encoded SUs [14,31]. Generally, the gene sequences of *accD* and *clpP1* (plastid genes) are relatively conserved among angiosperms but, in some independent lineages, evolution rates were found to be accelerated (quoted by [14]). Nearly all the nuclear encoded SUs of the Clp complex of the fast-evolving *Silene* species showed signatures of positive selection [14,20]. The greatest increase in substitution rate for the Clp complex was observed in the nuclear-encoded SU that is in direct interaction with the plastid SU. There was an enrichment of substitutions in the protein domain intimately interacting with this SU [14]. This pattern was less striking for ACCase, as *accD* does not evolve as fast as *clpP1* in the fast-evolving lineages. Thus, coevolution between nuclear-encoded and plastid-encoded SUs of Clp and ACCase complexes is suggested by signature of positive selection for compensatory mutations in *Silene* nuclear genes of these complexes [14]. Williams et al. (2019) conducted a study on the Clp complex across a broad range of angiosperms and observed correlated rates of accelerated evolution in the plastid-encoded and nuclear-encoded SUs [31]. Given these results, nuclear genes encoding SUs of plastid complex seem to experience positive selection to maintain coadaptation with their fast-evolving plastid genes.

A study on plastid ribosomes in Geraniaceae species found that signs of positive selection were detected on plastid genes, while the nuclear genes exhibited relaxed purifying selection [15]. Both nuclear and plastid genes exhibited accelerated $d_{N}$ compared to the rest of the genes studied (i.e., nuclear genes encoding other functions, including cytosolic ribosomes), but no sign of accelerated $d_{S}$ suggesting again that increased substitution rate was independent from the background mutation rate [15]. Here, the coevolution pattern seems to work in the opposite way and is unlikely to be due to the compensatory evolution of the nuclear interacting genes. This highlights the diversity of cytonuclear coevolution patterns [15,32]. Coevolution signals in plants were mostly found in protein complexes involving plastid genes with accelerated evolution, such as genes encoding essential plastidic factors (*ycf1* and *ycf2* [29]), RNA polymerase SU, ribosomal proteins [18], *accD* [14] and *clpP1* [28,31]. Concerning the genes encoding components of the photosynthetic apparatus, this coevolution pattern was less evident, due to their high sequence conservation and low rate of sequence evolution [11,12,14,28].

Similar studies on the possible interaction between mitochondrial and nuclear genomes are scarce, due mainly to the low mutation rate of the mitochondrial genome in plants [21]. However, a study on Oxidative Phosphorylation (OXPHOS) complexes, for which SUs are encoded by both mitochondrial and nuclear genes, was conducted on *Silene* by taking advantage of the occurrence of species with fast-evolving mitochondrial genomes. These fast-evolving species exhibited faster sequence evolution for the nuclear genes encoding SUs of OXPHOS complexes, in response to higher mitochondrial mutation rates, indicating a strong coevolution signal between nuclear and mitochondrial genes of these complexes [33]. Positive selection was also detected in the nuclear genes encoding SUs of the OXPHOS complexes, while none was detected for the control genes (i.e., nuclear genes not targeted to mitochondria) [34]. In addition, the strength of this signal depended on the complex analyzed: the strongest compensatory signal was observed for the complexes with the highest proportion of mtDNA proteins (i.e., OXPHOS complexes III & IV) [33]. Mitochondrial mutation rate seems to play a role in determining the rate of sequence evolution of the nuclear interactors, imposing strong selection pressure for compensatory changes on nuclear-encoded proteins interacting with mitochondrial gene products [34]. This was further confirmed through the analysis of the sequence evolution of mitochondrial and nuclear OXPHOS genes of 84 eukaryotes with various mitochondrial mutation rates [35]. Interestingly, the structural analysis of the mito-nuclear pairs in *Silene* systems showed that the nuclear substitutions in fast-evolving *Silene* species were preferentially found at residues in direct contact with mutated mitochondrial residue, suggesting a structurally mediated coevolution [33]. Other studies identified a potential influence of protein residues contact on the coevolution pattern [14], but this influence was not always detected, like in the Geraniaceae studies [15,25]. Further analyses, including the structural modelling of organellar complexes, should be carried out to better understand the involvement of direct contact in shaping patterns of coevolution and rates of sequence evolution.

CN coadaptation can also be revealed when crosses disrupt it, as in the case of F2 hybrids from *Arabidopsis thaliana* crosses, where reduced germination capacity was observed for certain CN combinations [36].

### 2.2. Cytonuclear Incompatibilities

In isolated populations or different species, the divergence of gene sequences (in nuclear or organellar genomes) occurs through the independent accumulation of mutations. Coadaptation and coevolution are lineage-specific: each lineage will have nuclear and organellar combination with coadapted gene sequences. When hybridization occurs between these lineages, organelles are exchanged, breaking the intergenomic combinations and disrupting the coadaptation between genomes. These mismatches between genomes may lead to CN incompatibilities (CNIs) [16].

CNIs appear to be widespread among taxa and genera, between and within species [12,37]. They often lead to altered phenotypes due to impaired organellar functions [38] and affect several fitness-related traits, such as viability and fertility of the hybrids [39]. These CNIs are often expressed directly in F1 hybrids [9,10,37] and often lead to asymmetrical decrease in fitness in reciprocal crosses, i.e., hybrids having the same hybrid nuclear background but different cytoplasms [40]. To further confirm CNI, backcrosses are often conducted (i) to partially restore or further disturb the parental CN combination and generate striking phenotypes [41,42], but also (ii) to disentangle the cytoplasm effect from the maternal one [43].

#### 2.2.1. CNIs Are the Result of Disrupted Coadaptation between Organellar and Nuclear Genes

Several studies of hybrid incompatibilities and CNIs provide insight on the mechanisms at play. Most of them concern plastid-nuclear incompatibilities (PNIs), which have been frequently observed in flowering plants and often result in chlorosis/virescence or variegation [41], due to a decrease in photosynthetic function [37,44].

In *Pisum*, PNI was identified in hybrids from crosses between *P. sativum elatius* (wild type—VIR320) and cultivated peas lines [45,46]. Anomalies in leaf pigmentation and pollen inactivation, among others, resulted in a reduction of hybrid fitness [45]. This reduction was asymmetric: almost all of the F1 hybrids were sterile and displayed chlorophyll deficiency when VIR320 was the maternal parent, while hybrids had normal phenotypes when VIR320 was the paternal parent [45]. Bogdanova et al. (2009) identified two unlinked loci potentially involved in this PNI: *Scs1* and *Scs2* [46]. Thus, for *Pisum* species, PNI was likely due to plastid complex malfunction (i.e., hybrid bleaching) resulting from a disrupted coadaptation.

PNIs can also emerge from other mechanisms. Many of the organellar genes require RNA editing (RNAe) of their transcripts which usually concerns C to U conversion [47]. RNAe sites can be species-specific [47,48] and the majority of the RNAe factors are pentatricopeptide repeat (PPR) proteins, encoded by nuclear genes and targeted to edit specific organellar genes [49]. Albino cybrids (i.e., ‘artificial’ hybrids generated by protoplast fusion and combining only one parental nuclear genome with only one plastid genome of another parent [44]) containing the nuclear genome of *Atropa belladonna* and the plastid genome of *Nicotiana tabacum* (i.e., Ab(Nt) cybrids) were reported [48]. Albinism seems to be due to a defect in RNAe of the transcript of *atpA* (plastid-gene encoding SU of the ATP synthase complex). In the Ab(Nt) cybrids, RNAe does not occur, due to the lack of specific RNAe factors in the *A. belladonna* nuclear genome, leading to impaired function of this complex [48]. CNI through editing disruption could also involve mitochondrial complexes, as editing is thought to be an important feature of the mitochondrial transcriptome [50].

Coevolution between organellar and nuclear genomes can also be disrupted in polyploids. Polyploidy results from nuclear whole genome doubling, leading to an increased number of nuclear genome copies. This phenomenon could disrupt CN interactions due to stoichiometry imbalance [51]. Allopolyploidization could make the maintenance of CN interaction even more challenging, as nuclear genome doubling results from hybridization. The allopolyploid individuals will thus contain biparental nuclear chromosomes and uniparental organellar genome inherited from different species, potentially resulting in CNI [51]. Several mechanisms are hypothesized to maintain coordination between organellar and nuclear genomes (reviewed in [51]): down-regulating the expression of the nuclear genes targeted to organelles (of both nuclear genomes or by the preferential expression of the organellar donor), or up-regulating the expression of organellar genes [52]. The study of allopolyploidization influence on CN interactions has mostly been studied on the Rubisco-encoding genes that revealed that maternal genes were preferentially expressed (reviewed in [51]). More recently, De Carvalho et al. (2019) studied the effect of recent and past allopolyploidization on the cytonuclear coadaptation in *Brassica*. In this study, no preferential transcription was identified. Rather, nuclear genes encoding SUs involved in plastid complexes were retained in duplicate or triplicate copies, while the genes encoding for cytosolic proteins were mostly found in single copy. Here the maintenance of CN coadaptation in allopolyploid *Brassica* seems due to the retention in multiple copies of the nuclear genes involved in plastid complexes, without any clear explanation of why the retention of multiple copies prevents CN maladaptation [13]. However, this study highlights the interest of conducting such approaches on a larger set of plant species, in order to have a better understanding of the consequence of polyploidization on CN interactions, polyploidization being an important feature of plant diversification [53].

#### 2.2.2. Acceleration of Organellar Genome Evolution Enhances the Propensity of CNIs

Accelerated rate of plastid evolution increases the propensity for CNI as it leads to a faster coevolution of organellar and nuclear genomes within populations [20,54,55]. Indeed, as nuclear and plastid genomes are coadapted and in tight coevolution, an increased rate of nucleotide substitution accelerates the local coevolution of these two genomes [28]. The variation in evolution rates of organellar genomes results in asymmetries in CNI: CNI will be stronger when the organellar donor comes from the population with the highest relative rate of organelle evolution [10].

*Campanulastrum americanum* exhibits accelerated evolution of its plastid genome [28]. A former study identified a plastid gene of the small plastid ribosome SU as a good candidate for the generation of PNI, as it exhibited elevated levels of NS substitution and its interacting nuclear gene encoding a SU of the same complex showed evidence of compensatory substitutions [28]. Post-zygotic reproductive isolation (RI) was observed between isolated populations, along with chlorotic hybrids (exhibiting chlorophyll deficiency), suggesting the presence of PNIs [28,41]. Further investigations of RI between populations of *C. americanum*, examining fitness-related traits in F1 hybrids from intra- and inter-clades crosses and backcrosses, demonstrated a reduction in survival, germination and pollen viability for chlorotic plants [41]. RI was asymmetric and dependent on the direction of the crosses and backcrosses, for all the measured traits [41]. The plastid genetic distance seemed to determine the strength of the RI: even if the populations were in a narrow geographical area, hybrid fitness decreased more when hybrids originated from more genetically divergent populations [56]. The accelerated rate of plastome evolution, influencing plastid genetic distances between populations, drove the evolution and strength of PNI [28,55,56]. Male sterility was also observed among Mountain lineages of this species, with a probable cytoplasmic contribution [56]. This kind of cytoplasmic-induced male-sterility is a phenomenon known as cytoplasmic male-sterility (CMS) and is the result of a conflict between the nuclear and mitochondrial genomes [7,57,58].

#### 2.2.3. CNIs as the Result of Intergenomic Conflict—the CMS Case

CMS is often the result of intergenomic conflict between the mitochondrial and the nuclear genomes [8,57,58]. Male-sterility mitochondrial genes will be selected as soon as they favor their own transmission via better seed production (their only way of transmission). This can be reached if the energy not devoted to pollen production is allocated towards seed production. Selection pressure will act on the nuclear genome for the emergence and fixation of the nuclear restorer of fertility (*Rf*) to re-establish the transmission through the pollen, by blocking the spread and expression of the CMS genes [59]. While this can lead to sexual polymorphism in populations, with the co-occurrence of hermaphrodites and females [60,61], CMS and *Rf* loci can also spread to fixation within a population, leading to ‘cryptic’ CMS, all individuals being hermaphrodite [62,63]. In this case, CMS will only be revealed in crosses involving populations that do not contain the proper nuclear *Rf* locus [62,63]. Thus, in case of hybridization between populations/species, CNI can be the result of CMS-*Rf* systems disruption among hybrids containing mismatched CMS genes and nuclear *Rf* [12,64]. *Rf* genes are often part of the PPR gene family which encodes proteins targeted to organelles and involved in organellar biogenesis, transcription regulation and RNAe [65].

In flowering plants, the mitochondrial genome is very fluid in terms of structure as it varies in size, non-coding and repetitive DNA content, and structural rearrangement [66]. CMS often arises from these frequent structural rearrangements, which produce chimeric open-reading-frames (*orf*) co-transcribed with essential genes [67]. These ORFs will be directly toxic for pollen or lead to reduce mitochondrial function, compromising the high energy demands of pollen development [40]. Nuclear *Rf* acts by post-transcriptional processing of these chimeric *orf* transcripts or proteins [40]. As most of the flowering plants are hermaphrodite, male sterility seems to be rarely expressed, even if CMS genes are likely common (i.e., often observed in interspecific crosses) [40,65]. It results in various outcomes, ranging from complete failure to develop male floral organs to arrest of pollen development at different stages [68].

Several cases of cryptic CMS have been identified and in particular the well-studied case of the *Mimulus* species. In hybrids between species of this genus, one-fourth of F2 hybrids bearing *Mimulus guttatus* cytoplasm were male sterile, while F2 hybrids bearing the cytoplasm of *M. nasutus* were all male fertile [40]. Crosses revealed that male fertility could be restored by the dominant allele of *M. guttatus* at a single locus. Under the hypothesis that CMS genes are co-transcribed with essential genes and that *Rf* genes modify the sequence size and/or stability of mt transcripts associated with sterility, *nad6* was identified as a potential candidate [65]. Further analyses suggest that the *Rf* locus identified in *M. guttatus* is composed of two linked loci: *Rf1* and *Rf2* both occurring in a cluster of tandemly repeated PPR genes, both dominant in this species [69]. They could act in two ways: (i) cleave the transcript portion associated with the sterility and prevent its expression or (ii) alter the transcription of start sites to prevent the production of longer transcripts potentially containing the CMS genes [65]. The sterilizing cytoplasm is present in only one population of *M. guttatus*, suggesting that: (i) this CMS has a limited dispersion, (ii) this population is where the CMS arose (iii) the coevolution of CMS and *Rf* genes was local [65]. Consistent with this hypothesis, the two *Rf* loci identified exhibit several signs of localized selection (i.e., distinct haplotype structure, signs of selective sweep in the *Rf* region containing these loci…) [62]. Mitochondrial CMS loci and their nuclear restorers seem to have coevolved, under strong positive selection and according to a model of conflictual coevolution, leading to a highly localized CMS-*Rf* system [62]. Another well-studied case of cryptic CMS concerns *Arabidopsis thaliana*, for which former studies identified CMS in crosses between two distant accession: Sha and Mr-0 [70,71]. The F1 hybrids plants containing Sha cytoplasm were sterile and unable to produce pollen, while F1s with the Mr-0 cytoplasm produced viable seeds [71]. The Sha lineages appeared to contain CMS factors and the nuclear *Rf* to restore male fertility [70].

CNI can arise through the disruption of coadaptation in major organellar complexes and intergenomic conflict between mitochondrial and nuclear genes. Much still needs to be done in order to understand the mechanisms of coevolution between genomes and which cellular functions are mainly affected by disruption of coadaptation. Databases on protein-protein but also RNA-protein or DNA-protein interactions could be useful to further investigate and identify nuclear and organellar interacting partners, coupled with the analysis of whole organellar genome diversity and functional validation [72]. The calculation of CN linkage disequilibrium (i.e., cnLD, representing the non-random association of organellar and nuclear alleles) could also help identify coadapted couples of nuclear and organellar genes [73].

### 2.3. Cytonuclear Coevolution and Environment

In animals, mitochondrial genomes can be involved in adaption to climate or altitudinal variation [74]. A growing number of studies are focusing on the influence of cytoplasmic variation and its potential impact on environmental adaptation in plants (reviewed in [12,75]), and point out the potential role of organellar genomes in local adaptation.

Transplantation experiments were conducted with two *Helianthus* species, living in mesic (*H. annuus*) or xeric (*H. petiolaris*) habitats. They revealed that the cytoplasms of these species were adapted to each specific habitat, suggesting that variation in the organellar genome could contribute to local adaptation and ecological differentiation [76]. Potential cytoplasmic introgression driven by selection among *Helianthus* species was also reported, leading to widespread CN discordance in genealogies [77]. Selection might shape the pattern of organellar variation for some specific genes, resulting in adaptive introgression of the plastid genomes favoring local adaptation [77]. Cytoplasmic genomes can be important capacitors for the generation of novel phenotypes in specific environments, as shown in a study on *Arabidopsis thaliana* [42]. In addition, in this species, several adaptive traits seemed to be influenced and even shaped by CN interactions (around 80%) and organellar genome variation [38]. In *A. thaliana*, germination experiments on 64 cytolines were conducted among populations from different geographical locations. A significant effect of the cytoplasm genotype on seed germination efficiency was identified, suggesting a role of the selection on the plasmotype geographical distribution and its role in *A. thaliana* populations adaptation [36]. Another study conducted on the same cytolines concluded that cytonuclear interactions and coevolution had an impact on adaptive traits linked to seed vigor [78]. For example, the creation of novel CN combinations in cytolines, leading to the disruption of CN coadaptation, had a deleterious effect on adaptive dormancy and germination, consistent with a contribution of CN co-adaptation and cytoplasmic variation on complex traits involved in plant adaptation [78].

Plastid genes encode components of essential functions in plant cells such as photosynthesis, a process that is influenced by many environmental factors and often associated with fitness differences [12,37,38]. The extreme degree of conservation of the genes encoding photosynthetic apparatus suggests that they evolve under genetic drift and/or strong purifying selection [11,12]. Yet, positive selection was identified for some specific genes and in particular lineages, pointing out a potential role of these genes on local adaptation [11,12,31]. For example, positive selection was found in the photosynthetic *rbcL* plastid gene, encoding a SU of the essential Rubisco enzyme [12,77]. Another study reported that the nuclear genes involved in photosystem I (PSI) had a very low absolute rate of substitution (as was expected given that this complex is slowly evolving), but also a significant excess of NS divergence between lineages. This demonstrates that some NS substitutions could be adaptive and spread under positive selection rather than fixation through genetic drift [14]. Positive selection for certain plastid genes was also discovered in the *Helianthus* species, suggesting the adaptive value of these genes [77]. In the peculiar case of anthropogenic environments, the plastid was directly involved in the adaptation to herbicide: a unique substitution in plastid *psbA* gene conferred resistance to Triazine in wild populations of *Arabidopsis thaliana* [79]. Selection could shape plastid sequence variation, depending on environmental factors and drive coevolution pattern between locally adapted plastid genes and the nuclear counterparts. These genes could also have adaptive value in relation to photosynthetic performance and colonization ability (see [76,79]).

If such a relation between organellar variation and local adaptation exists, then it could also drive the emergence of CNIs between locally adapted genes [76]. Indeed, specific environment can generate specific conditions leading to the selection for a particular mutation in the plastome and favor the compensatory mutation in the nuclear partner. This would lead to CN coadaptation driven by environmental conditions and the creation of CNIs among hybrids from populations coming from different environments [12]. In *C. americanum*, PNI could be due to local adaptation of one particular clade: reduced germination was observed in all crosses involving this clade, while there was none when it was absent from the crosses. This clade is geographically and environmentally distinct from the others and thus, its local adaptation could particularly contribute to disrupted CN coadaptation and PNI [41,56].

### 2.4. CNI Can Contribute to Speciation

Several factors can impulse the generation of CNIs, such as an accelerated rate of organellar genome evolution or the involvement of organellar genomes in local adaptation [9,28,40]. As CNIs often result in reduced survival or germination and hybrid breakdown, it is very likely that both organellar genomes and CNI play a role in speciation through the development of barriers to gene flow between lineages [9,36,37]. CNIs are part of the genetic incompatibilities fitting the BDMI model [9,37,41] and are thought to be among the earliest genetic incompatibilities to arise in speciation, and play an important role in the emergence of post-zygotic barriers to reproduction [9,16,37,40,55,76]. Their contribution to such a process depends on several factors, such as the evolutionary history of the interaction (i.e., selfish cytoplasmic coevolution or neutral/adaptive coevolution), genetic characteristics of the parental species and the loci under consideration [32,40]. The strength of post-zygotic barriers due to CNIs also appears to be strongly associated with the degree of cytoplasmic divergence [9,37].

The possible involvement of CMS in post-zygotic reproductive isolation is still unclear [5,7,12]. Due to its strong initial impact on individual fitness, CMS could represent a barrier to introgression, even more if the CMS-*Rf* system is geographically localized and when sterility is selected against in hybrids [40]. However, this effect will be short term, as the cost of male sterility might not be high enough to prevent the introgression of a CMS cytotype (conferring a better seed production to females) into a non-CMS population [62]. CMS participation in RI is even more unlikely if the matched nuclear restorer is also transmitted, as this system could increase introgression in secondary hybridizing populations [40]. Nevertheless, CMS-*Rf* systems can lead to a rapid genetic divergence between populations: a selective sweep due to CMS mutation will carry associated mitochondrial variation and impact mitochondrial divergence between populations, with consequences for organellar function. In the coadapted nuclear *Rf*, the responding sweep will alter the dynamic of linked nuclear variation and promote divergence between populations for relatively large regions of the nuclear genome [62]. Thus, local CMS-*Rf* dynamics is likely to generate species-wide incompatibilities and contribute to the establishing post-zygotic barriers during the early stage of speciation; even more so if its emergence is linked to specific ecological conditions [62,65]. Differences in mating system between the two hybridizing species and the fitness costs of the nuclear restorer alleles, depending on environment, could enhance the impact of this kind of CNI on the speciation process [62].

The nuclear component of CNIs could also play a role in speciation process. For example, the nuclear genes potentially involved in PNIs between *Pisum* exhibit high variability between lineages, even within the same geographical area [80]. This variability leads to different degrees of incompatibility and these genes could be viewed as ‘speciation genes’ [80], i.e., genes involved in RI as they contribute to the gene flow barrier between lineages [13].

The potential role of cytoplasmic variation and CN interactions in local adaptation could be an important driver of speciation, as it could maintain species-specific ecological differences [76]. This would generate associations between genes involved in ecological divergence and genes contributing to maladaptive CN interactions in hybrids. These associations would facilitate the origin and maintenance of species in presence of gene flow [76]. Ecological selection on a cytoplasm should limit its introgression and the introgression of the nuclear alleles interacting with it, maintaining the phenotype of the species despite hybridization [76].

Multiple genetic incompatibilities generally play a role in the reproductive isolation for several traits, during the entire plant life cycle [41,81]. For example, in *C. americanum*, the number of incompatibilities and the strength of the reproductive isolation is variable depending on the lineages crossed. The post-zygotic reproductive isolation identified in *Mimulus* hybrids (*M. guttatus* × *M. nasutus*) seems to be controlled by different hybrid incompatibilities, also depending on the populations crossed [81].

Organellar genome pattern of inheritance could impact CN dynamics and the role of CNIs in speciation. Indeed, biparental inheritance could be selected to avoid CNIs and reduce the involvement of CNIs in speciation.

## 3. Can Pattern of Organelle Inheritance Influence CNIs?

### 3.1. Inheritance Patterns of the Organellar Genomes

Unlike the nuclear genome, inherited biparentally and following Mendelian segregation, the organellar genomes are mostly uniparentally inherited, usually maternally [19]. Uniparental inheritance can lead to severe evolutionary consequences (see Section 2.1), but also confer several evolutionary benefits. For example, it will favor the avoidance of deleterious interactions between co-existing organelle genomes potentially leading to disruption of CN coadaptation [12,19,37]. It will also limit within individual organellar diversity, since organellar genomes will go through a genetic bottleneck in the germline. This will limit the spread of selfish elements (mitochondrial) and lead to homoplasmy and the elimination of malfunctioning genotypes by selection [19,82]. The predominance of maternal inheritance remains largely unclear. It is likely due to the higher mutational load in the paternal gamete (the smaller one), more severe oxidative damages and more pronounced genetic drift, especially if the number of organellar DNA copies in the sperm cell is small. This mutational load will favor the evolution of gamete-controlled organelle exclusion mechanisms (“Killing one’s own cytoplasm”) and will result in maternal inheritance of organellar genomes [19].

Uniparental transmission of organelle genome has been repeatedly lost and restored over evolutionary timeframes. Mutational meltdown can be erased temporarily or over longer periods of sexual recombination between organelles through biparental transmission [19].

### 3.2. Heteroplasmy: Evidences and Consequences

Biparental transmission has arisen multiple times within the angiosperms and about 20% of the angiosperm species have the potential for biparental inheritance [55,64,83]. Accidental paternal transmission of organellar genomes (i.e., paternal leakage) can occur in crosses of divergent populations or species, due to a breakdown of mechanisms preventing biparental transmission [19,64,82]. In this case, paternal organellar DNA can endure different fates: it can be (i) destroyed within the sperm cell, (ii) physically excluded from the egg cell during fertilization or (iii) successfully replicated and maintained in the zygote after fertilization [39,64,82]. The latter case will lead to heteroplasmic individuals with two organellar haplotypes. Evolutionary consequences of heteroplasmy through paternal leakage will depend on the level of heteroplasmy (the ratio of paternal/maternal cytoplasms) at which the organellar genome has been paternally transmitted, but also the context in which it occurs. We can expect that, if the paternal organellar genome is in low frequency, it has a good chance of being lost during cell multiplication and organellar sorting out. Conversely, substantial levels of heteroplasmy may provide sufficient genetic variation for selection to act upon. In the case of mitochondrial genomes, it can even lead to genotypic novelty via recombination. In the case of paternal leakage of the plastid genome, the two plastid genomes will not fuse and thus will not undergo recombination. This will lead to competition between the two parental plastids. Their ability to compete against each other partially depends on the lipid composition of the plastid membrane, as it determines plastid stability and division rate [83].

In hybrids resulting from crosses between divergent population or species, biparental inheritance and the paternal leakage of plastids can result in leaf variegation. Hybrids will have variegated leaves or fully green/white ones, as a result of sorting-out the two plastid types during ontogenesis [83,84]. It indicates that one of the two parental plastids is unable to develop and undergo normal differentiation under the hybrid nuclear background, potentially because it is incompatible, while the other one is not [37,84,85,86,87], leading to chlorophyll-deficiency leaf sectors (i.e., white or yellowish sectors) [64,85].

Evidence of heteroplasmy and variegation exists in literature. For example, a study of Yao et al. (1995) identified that crosses between *Zantedeschia odorata* and two other species (*Z. elliottiana* and *Z. aethiopica*) resulted in hybrid variegation [88]. Heteroplasmy and hybrid variegation was also observed from crosses with *Pelargonium* species [84]. The analysis of the parental origin of the plastids in the green and white sectors of the variegated hybrids leaves revealed that in the green sectors, plastid DNA (ptDNA) of *Pelargonium zonale* *‘Roseum’* was present, while ptDNA of *P. zonale hort. ‘Stadt Bern’* was present in the white tissue [84]. This suggests heteroplasmy and sorting out of the plastid types, the *‘Stadt Bern’* one being incompatible with the hybrid nuclear background. Weihe et al. (2009) also analysed the inheritance pattern of both organellar genomes in the progeny of reciprocal crosses between *P. zonale* × *P. inquinans*. They observed biparental transmission of the two organellar genomes and hybrid plants exhibited variegation with the *P. inquinans* plastid bleaching out, potentially due to its incompatibility with the hybrid nuclear genome [85].

### 3.3. Paternal Leakage Rescues from Cytonuclear Incompatibilities

While hybridization between species can lead to biparental transmission of organelles and result in variegated hybrids, biparental transmission can also favor the restoration of compatibility between the plastid and nuclear genomes, leading to hybrid fitness recovery [64]. This rescuing could be due to several reasons. First, in crosses resulting in CNIs, occurrence of biparental inheritance could increase the likelihood that hybrids inherit an organellar genome compatible with the hybrid nuclear background. Second, as biparental transmission introduces genetic variation among organelles, selection can occur and could lead to the loss of the incompatible organellar genome [55]. Biparental transmission also seems to maintain the linkage disequilibrium between compatible cytoplasmic and nuclear genomes (cnLD) and thus favor the maintenance of adapted combinations between nuclear and organellar genomes [73]. Ramsey et al. (2019) showed that in gynodioecious species *Daucus carota*, hybrids from two distant populations exhibited lower fitness when parents were homoplasmic than when they were heteroplasmic [73]. Heteroplasmy could mitigate the negative effects of CNIs and maintain individual fitness, by providing cytoplasmic alternative allelic variants that potentially ‘match’ better with nuclear alleles [73].

Paternal leakage and heteroplasmy frequently occur in taxa that exhibit CNIs. Several studies showed that biparental inheritance could indeed increase the fitness of hybrids experiencing CNIs [55]. In crosses between *Pisum sativum* ssp. *elatius* wild species and cultivated peas forms, hybridization resulted in hybrid variegation. Paternal leakage occurred in crosses associated with PNI (i.e., chlorophyll deficiency): the fully green sectors of the variegated leaves contained paternally inherited plastids, suggesting that paternal plastid genome led to the recovery of normal photosynthetic performance [87,89]. Concerning the mitochondrial genome of pea, it appeared to be of maternal origin, indicating that paternal leakage was potentially driven by the presence of PNIs in hybrids [87].

Crosses in *C. americanum* revealed that biparental inheritance was constitutive in the species and did not depend on the level of genetic divergence between populations [55]. Biparental transmission led to an increased survival of F1 hybrids by enabling the selection against the incompatible plastid genome [55]. As F1 hybrids with heteroplasmy displayed better fitness, they contributed to the majority of surviving F2 hybrids, which enforced the loss of the incompatible plastid. This phenomenon was stronger in crosses between divergent clades, suggesting that the level of CNI triggered the hybrid fitness recovery [55]. This phenomenon could lead to a counter-intuitive result: since CNI is stronger for hybrids from crosses between isolated populations, it could favour introgression and lead to the collapse of reproductive isolation through heteroplasmy. Paternal leakage and heteroplasmy could thus slow down the speciation process, by rescuing from strong incompatibility, resulting in weaker reproductive barriers [55]. Given this, accelerated plastid genome evolution, which enhances the propensity for PNI, could also indirectly influence the level of paternal leakage [55]. Interestingly, several taxa exhibiting biparental inheritance also exhibit accelerated rates of plastid evolution [28].

CN interactions could regulate the paternal organellar DNA transmission and its selective replication in hybrids, in order to overcome CN dysfunction [39]. In a study of crosses between barley and wheat, Aksyonova et al. (2005) [39] reported the occurrence of a biparental transmission of organellar DNA and revealed a shift toward the paternal organelle DNA during repeated backcrosses. This shift indicated that paternal organellar DNA was transmitted through pollen and successfully replicated in the zygotes. Plastid transient heteroplasmy likely occurred as only paternal copies of wheat were detected in the stable self-fertile and vigorous lines obtained in the backcross generations. The increase in wheat paternal ptDNA content was correlated with fertility and restoration of vigor in these lines. Barley nuclear chromosomes were undetected and apparently replaced by wheat chromosomes. These results suggest that (i) the paternal wheat ptDNA was transmitted and selectively replicated, resulting in hybrid fitness recovery and (ii) that the wheat nuclear genome encoded for effective replication and retention of its corresponding ptDNA. Most likely, repeated backcrosses with wheat parents led to the replacement of the nuclear barley chromosomes and promoted the selective amplification of the paternal wheat organellar DNA copies [39].

## 4. How Could Mating Systems Favor or Limit CNI?

Mating systems are known to shape intra- and interspecific genetic variation [90], affect the efficacy of selection [91] and introduce selective forces in hybrid zones, where RI is incomplete [92]. The emergence of post-zygotic barriers to reproduction can be influenced by disruption of CN coadaptation. Studies are emerging about the impact of mating systems on the maintenance of coadaptation between interacting partners from different cellular compartments.

Mating systems can influence the degree to which cytonuclear allele combinations are inherited [93]. For example, selfing rate increases the heritability of CN allele combinations. The higher the selfing rate, the more CN allele combinations will tend to be inherited as single units [93]. This will allow direct selection of CN allele combinations, favoring the beneficial and eliminating the deleterious ones. Selfing will be more efficient than random mating or separate sex species [93]. Therefore, it is expected that crosses between distant populations of selfing species will disrupt such co-adapted CN units and thus generate strong CNIs. Studies on crosses in selfer *Arabidopsis thaliana* are in concordance with this expectation [36].

Transfer of organellar genes to the nuclear genome could also impact the level of CN coadaptation. Such transfers will not have the same probability of occurring, with regards to the mating system of the species. Selfing species will have an increased rate and probability of gene transfer compared to an outcrossing species, even more so if the transfer is adaptive [93,94]. In highly selfing species, the loss of the functional duplicated organellar copy will have no deleterious fitness consequences, as the nuclear functional gene copies will remain associated with the organellar genome. However, for outcrossing species, the organellar genome lacking the duplicate gene function will have a selective disadvantage compared to genomes with functional copies, since its function must be rescued by ‘association’ with a functional nuclear copy. In this context, as interspecies hybridization between an outcrosser and a selfer species will preferentially imply that the outcrosser is the pollen donor [95], it is expected that hybrids will exhibit CNIs, since the nucleus from the outcrosser will lack the gene(s) missing on the selfer organellar genome.

The presence of sex chromosomes may further reinforce genetic conflicts that might lead to segregation distortion [92]. Segregation distorters and their suppressors coevolve independently within a species and their interactions in hybrids could be a source of CNIs [92]. A shift in nuclear chromosomal distribution of the nuclear genes encoding gene products targeted to the organelles could also be observed, to maintain CN coadaptation (quoted in [96]). This chromosomal distribution is thought to be influenced by the inheritance pattern of organellar genes [96]. Male sex-chromosomes are often heterogametic (i.e., XY) and the female ones, homogametic (i.e., XX). Thus, the genes located on the X-chromosomes spend 2/3 time in females. Organellar DNAs are co-transmitted with 1/2 of the autosomal genes, 2/3 of the X-linked ones and none of the Y-linked ones [17] As X-linked genes have a higher probability of co-transmission with organellar genes, selection for coadaptation could result in the overrepresentation of nuclear interacting genes on X chromosomes compared to the others [17,96]. In cases of sex-specific CN coadaptation disruption, this could induce chromosomal incompatibilities following Haldane’s rule [97] (i.e., reduced fitness of the heterogametic sex in hybrids between species or divergent populations [10,98]). Another theory exists, named the sexual conflict hypothesis, for which the opposite chromosomal distribution is expected: more CN interactions involving autosomes to reduce the mutational load in males [99]. So far, in the only plant case studied (*Rumex hastatulus)*, no evidence for over- or under- representation of genes interacting with organelles on X chromosomes has been found [96]. This could be due to the relatively young age of its sex-chromosomes, leaving no time for selection to act on coadaptation or sexual antagonism [96]. The development of new protocols for sex-determination on non-model species should enable to tackle these hypotheses on a larger scale, with a gradient of sex chromosome age [100].

Last, CNIs could impact the evolution of mating systems. In particular, in certain conditions, CMS can generate gynodioecy (females and hermaphrodites in populations) that can be seen as a stable mating system over time through balancing selection, an evolutionary dynamic that maintains old cytoplasms. This could favor the accumulation of genetic incompatibilities on organellar genomes, as suggested in the gynodioecious *Silene nutans* which revealed cryptic speciation [101,102]. Interestingly, gynodioecy can also be a transitory step towards separate sexes (dioecy) [103,104].

Box 1 **Acceleration pattern and selective forces acting on the organellar genomes.**

Acceleration pattern is expressed as an increased rate of nucleotide substitution and an excess of non-synonymous (NS) substitutions compared to synonymous (S) ones, leading to an elevated $d_{N}/d_{S}$ (annotated ω, with $d_{N}$ as the NS substitution rate and $d_{S}$ as the S substitution rate). This acceleration can reveal either positive selection or relaxed purifying selection, due to the reduced Ne of the organellar genome [14,20,28]. Determination of selection forces generally relies on the estimation and analyses of *ω*: ω < 1 indicates purifying selection, ω=1 neutral evolution, ω>1 indicates positive selection. It is an effective way to disentangle the effect of higher mutation rate or changes in selective pressure. S substitutions being considered as neutral, $d_{S}$ likely reflects the underlying mutation rate, while $d_{N}$ is impacted by the underlying mutation rate and selection [28]. Thus, changes in $d_{N}$ gives insight into changes in selection [28]. Other statistics exist for the detection of positive selection, such as the Tajima’ D and the Fu’s $F_{s}$ which are useful to detect deviation from neutral variation or the McDonald and Kreitman test (MKT), which specifically tests for positive selection [77].

Distinguishing between positive selection and relaxed purifying selection is difficult, as they lead to the same pattern of increase of $d_{N}/d_{S}$ ratio [31]. Looking at the variation of intra- and interspecific sequences can be a promising approach, since in the case of positive selection, the fixation of NS substitutions leads to an increase in ω between species (i.e., interspecific divergence), compared to intraspecific polymorphism [18]. It must be noted that molecular tests for selection detection face several limits, for example limited power if the sequence variation is low and thus the inability to perform such tests on very closely related taxa [77]. Moreover, in the majority of these tests, $d_{S}$ is the reference against which selection is measured, but selection could also occur on S substitutions, leading to a potential contribution of these substitutions on differential fitness between individuals [77]. A molecular test can be used to detect relaxed selection on molecular sequence data: RELAX [105]. It has often been used in coevolution studies [15,31,34] and is based on a comparative phylogenetic framework, comparing gene-wide selection intensity across phylogenetic branches (see [105] for details).

## Glossary

**Chlorosis**	loss of the green coloration of the plant leaves due to chlorophyll deficiency.
**Cytonuclear Linkage Disequilibrium (cnLD)**	represents the non-random association of nuclear and cytoplasmic alleles and is influenced by several factors, such as the inheritance pattern of organellar genome, hybridization, selective forces, mating system… Its evolutionary significance will depend on its magnitude and how stable CN interactions are [73,96].
**Effective size (Ne)**	the effective size of a population is a parameter that is used in population genetics to quantify the effect of genetic drift on the genetic diversity of a population. As the effective size decreases, the effect of genetic drift will be stronger and the efficacy of selection lower. This concept can be applied at the population level, but also at the genome or gene levels. For example, Ne is affected by the mode of inheritance: due to uniparental inheritance, Ne is lower for organellar genes than for autosomal ones which are transmitted biparentally [106].
**Functional gene transfer**	transfer of an organellar gene and its function to the nucleus. The new nuclear gene will be targeted back to the organelle and the redundant organellar gene will be eventually lost [94].
**Genetic drift**	Evolutionary process represented by the random sampling of alleles. It leads to changes in allele frequencies in a population over generation and influence population genetic diversity and divergence among populations.
**Heteroplasmy**	co-occurrence of two or more different organelle genotypes within an individual, which can be the result of biparental transmission.
**Intergenomic coevolution**	involves reciprocal effects of selection on interacting molecules from two genomes [16].
**Non-Synonymous substitutions (NS)**	single-nucleotide mutations leading to changes of amino-acid in the encoded polypeptide.
**Paternal leakage**	occasional transmission of paternal organellar genome.
**Synonymous substitutions (S)**	single-nucleotide mutations that do not result into changes of amino-acid in the encoded polypeptide.
**Variegation**	presence of different colors, from green to white, on sectors on the same leaves or on different leaves of the same plant.
